# Editorial on Special Issue “Gels for Oil and Gas Industry Applications”

**DOI:** 10.3390/gels8080513

**Published:** 2022-08-18

**Authors:** Qing You, Guang Zhao, Xindi Sun

**Affiliations:** 1 School of Energy Resources, China University of Geosciences (Beijing), Beijing 100083, China; 2 School of Petroleum Engineering, China University of Petroleum (East China), Qingdao 266580, China; 3 Physics and Engineering Department, College of Health, Engineering and Science, Slippery Rock University of Pennsylvania, Slippery Rock, PA 16057, USA

This Special Issue includes many advanced high-quality papers that focus on gel applications in the oil and gas industry. The papers in this Special Issue present the new development of gels that can be used as conformance control agents, drilling fluid additives, and hydraulic fracturing agents.

As common conformance control agents, gels have been applied in conventional reservoirs for decades. Recently, more and more research has focused on gel application in harsh environments, including high salinity and/or high temperature. Ding et al. [1] introduced the gelling behavior of PAM/Phenolic crosslinked gel and its profile control in a low-temperature and high-salinity reservoir. The results indicated that this gel could form a strong “stem-leaf”-shaped 3D network structure in deionized water. In addition, this structure remains stable in high-concentration salt solution. Therefore, this novel gel is suitable for conformance control of high-salinity and low-temperature reservoirs. Wang et al. [2] reported the enhanced oil recovery mechanism and technical boundary of gel foam for Changqing oilfield. In this study, the composite gel system and gel foam system were compared from the perspective of plugging performance and oil displacement performance. The results revealed that the composite gel system is stronger in terms of plugging performance, however, the gel-enhanced foam showed high oil displacement efficiency due to the “plug-flooding-integrated” feature of the foam. Qu et al. [3] studied the performance of soft movable polymer gel for water coning control of horizontal wells in offshore heavy oil cold production. The results indicated that this gel has a compact network structure and excellent creep property. The field application results revealed that the oil rate increased from 9.2 m^3^/d to 20.0 m^3^/d, the average water cut was reduced to 60–70%, and the cumulative oil production was predicted to increase almost 3-fold. Cheng et al. [4] used experiments and numerical simulation to investigate the oil displacement mechanism of weak gel in waterflood reservoirs. They concluded that weak gel selectively enters and blocks large channels and diverts subsequent water flow to the unswept area. In addition, the oil droplets converge to form an oil stream due to the negative pressure oil absorption created by the weak gel’s viscoelastic bulk motion. In addition to conformance control agents, gels can also be used as additives in drilling fluids. Tang et al. [5] reported novel nanoparticles (acrylamide/2-acrylamide-2-3 methylpropanesulfonic acid/styrene/maleic anhydride polymer-based nanoparticles), which can improve the filtration of water-based drilling fluids at a high temperature. The rheological properties of the drilling fluids that were treated with these nanoparticles were stable before and after aging at 200 °C/16 h and the filtration control was improved. As a consequence, this novel nanoparticle can improve colloidal stability and mud cake quality at a high temperature. Tang et al. [6] also studied the effect of novel catechol-chitosan biopolymer encapsulator-based drilling mud on wellbore stability. The rheology properties of this drilling fluid were stable before and after 130 °C/16 h hot rolling and the shale inhibition behavior is good. Additionally, the novel drilling fluid could chelate with metal ions and form a stable covalent bond, which improved the adhesiveness, inhibition, and blockage. Wei et al. [7] presented a salt resistance copolymer and its application in oil well drilling fluids. Compared with other small molecular copolymer filtrate reducers, this new copolymer has better resistance to complex salts, better filtration-loss-controlling performance, and better resistance to saturated brine. Long et al. [8] introduced a new drilling fluid filtrate reducer and reported its preparation and hydrogelling performance. Compared with commercial filtrate reducers, this novel filtration reducer shows good thermostability and salinity resistance. The filtration loss performance is comparable with a low viscosity sodium carboxymethyl cellulose and carboxymethyl starch. Gels are also an important component in hydraulic fracturing. Shibaev et al. [9] summarized the novel trends in the development of surfactant-based hydraulic fracturing fluids. This review paper described the novel concepts and advances of viscoelastic surfactant-based (VES) fracturing fluids published in the last few years. This paper covered the use of oligomeric surfactants, surfactant mixtures, hybrid nanoparticles, or polymer/VES fluids. The advantages and limitations of different VES fluids were systematically discussed in this paper. Wang et al. [10] presented a new compound staged gelling acid fracturing method for ultra-deep horizontal wells. Sichuan carbonate gas reservoirs are used as models in a simulator to predict the performance of this new staged gelling acid fracturing method. The simulation results indicated that the crosslinked authigenic acid and gelling acid in the Sichuan carbonate gas reservoir are injected alternatively in three stages and the total production was 2.1 times higher than the conventional acid fracturing. 
